# Modulators of Diacylglycerol Kinase Activity: A Review of Advances and Challenges

**DOI:** 10.1002/med.70010

**Published:** 2025-08-26

**Authors:** Luisa Racca, Gianluca Baldanzi, Alberto Massarotti

**Affiliations:** ^1^ Department of Translational Medicine Università del Piemonte Orientale Novara Italy; ^2^ Center for Translational Research on Allergic and Autoimmune Diseases (CAAD) Università del Piemonte Orientale Novara Italy; ^3^ Department of Pharmaceutical Sciences Università del Piemonte Orientale Novara Italy

**Keywords:** activators, clinical trial, DGK inhibitors, diacylglycerol kinase (DGK), drug design

## Abstract

Catalyzing the conversion of diacylglycerol (DAG) in phosphatidic acid (PA), diacylglycerol kinases (DGKs) play a pivotal role in all the physiological processes modulated by these two bioactive lipids, such as lipid metabolism and immune regulation. Consequently, abnormalities due to a dysregulation of DGK's activity are involved in several pathological contexts, from cancer to autoimmune diseases. Interestingly, ten DGK isoforms with specific structure and expression pattern are present in humans, suggesting nonredundant roles. Despite their potential as therapeutic targets, the possibility of selective DGK pharmacological modulation remains limited to two isoforms. However, the research for DGK isoform‐specific modulators is growing, as well as the interest in the structure and functioning of all DGK family members. This review aims to present all the information on DGK modulators, from the literature to patents' databases, starting from what we know about DGK's structure, the key physiological and pathological processes where they are involved and, above all, to understand which are nowadays the possibilities for DGK activation/inhibition. Our aim is to inspire future investigations which could accelerate the discovery of new DGK‐targeting compounds.

## 1 Introduction

Diacylglycerol kinases (DGKs) phosphorylate diacylglycerol (DAG, 1,2‐diacyl‐sn‐glycerol) to generate phosphatidic acid (PA, 1,2‐diacyl‐sn‐glycerol‐3‐phosphate). These two bioactive lipids play roles in both lipid metabolism and signal transduction. DGKs act as key enzymes in regulating cellular processes by maintaining a balance between DAG consumption and PA production. DAG activates and regulates signal transduction proteins with C1 domains, including conventional protein kinase C (cPKC), novel PKC (nPKC), Ras guanyl nucleotide‐releasing protein (GRP), and so forth. Otherwise, PA modulates several factors, such as mammalian target of rapamycin (mTOR) and atypical PKC. Contrary to the traditional belief that DGKs only process DAG derived from phosphatidylinositol turnover, recent studies have shown that DGK isoenzymes can process a wide variety of DAG species. These DAG species originate from various pathways, including sphingomyelin synthases and other proteins such as PHOSPHO1 acting as phosphatidylcholine‐specific PLC [1, 2].

Moreover, the sequential action of phospholipase D and PA phosphatases are a relevant source of DAG for signaling in epithelial cells [3], but also in lymphocytes at the Golgi level [4]. Those findings suggest that the localization domains in each isoform not only control membrane recruitment and activation but also restrict the action of each isoform to distinct DAG pools.

DGKs are therefore involved in several physiological and pathological processes, which explains the growing interest in understanding their structure, role in signaling and involvement in pathologies, but also the research efforts for developing pharmacological inhibitors.

**Figure 1:**
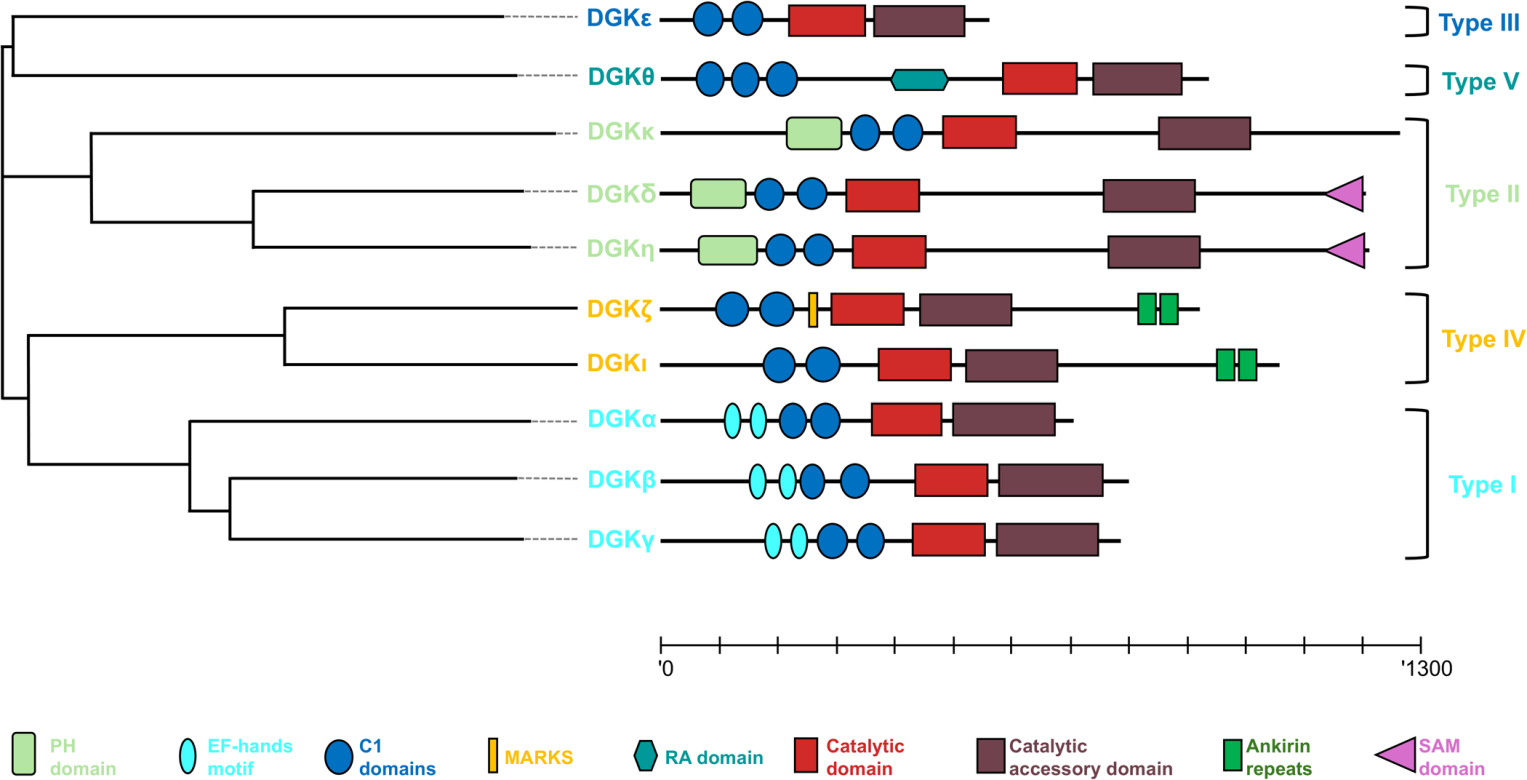
Phylogram with human DGKs from UniProt with their schematic representation based on SMART database [7] annotations on human canonical isoenzymes from UniProt (DGKα:P23743; DGKβ:Q9Y6T7; DGKγ:P49619¸DGKδ:Q16760; DGKη:Q86XP1; DGKκ:Q5KSL6; DGKε:P52429; DGKζ:Q13574; DGKι:O75912; DGKθ:P52824). Scale bar 1300 amino acids. [Color figure can be viewed at wileyonlinelibrary.com]

To date, 10 DGK different isoenzymes (Diacylglycerol kinase alpha: DGKα; Diacylglycerol kinase beta: DGKβ; Diacylglycerol kinase gamma: DGKγ; Diacylglycerol kinase delta: DGKδ; Diacylglycerol kinase eta: DGKη; Diacylglycerol kinase kappa: DGKκ; Diacylglycerol kinase epsilon: DGKε; Diacylglycerol kinase zeta: DGKζ; Diacylglycerol kinase iota: DGKι; Diacylglycerol kinase theta: DGKθ) have been described in mammals, but the presence of different splicing isoform, such as ζ1 and ζ2, expands the diversity of this family, which has only been partially understood so far. Based on sequence homology and structural features, DGKs are classified into five groups: Type I includes α, β, and γ; Type II contains δ, η, and κ; Type III includes only DGKε; Type IV comprises ζ and ι; and Type V consists solely of θ. The presence in every isoform of at least two equally spaced cysteine‐rich domain (C1) in close proximity to a split kinase domain (consisting of a C‐terminal catalytic and an accessory domain), suggests a common phylogeny for the family. There are also several subtype‐specific functional domains mainly located at the N terminal [5, 6] that putatively confer isotype‐specific function and regulation (Figure 1).

## 2 Different Isoforms With Different Features

While the integral membrane bacterial DgkA is a well‐characterized protein [8], it is evident that there is little information in the literature on 3D structure of mammalian soluble DGKs, primarily due to the challenges associated with obtaining sufficient amounts of homogeneous purified protein [9]. To date, we possess only partial structural information about human DGKα [10, 11] δ [12, 13] and ζ [14] that can be integrated with tools as AlphaFold, a protein structural prediction technology powered by artificial intelligence [15, 16]. Additional insights can be gained from the study of prokaryotic DGK‐related enzymes [9], such as the *Staphylococcus aureus* DgkB [17]. All the information disposable for human DGKs, both from 3D reconstruction and prediction, is listed in Table 1.

**Table 1:** Experimental and predicted 3D structures for all the 10 human DGKs available in Protein Data Bank (PDB) on July 2025.

Isoform	Uniprot code	Code	Method	Positions
DGKα	P23743	1TUZ	NMR spectroscopy	1–116
		6IIE	X‐ray crystallography	107–197
		AF‐P23743‐F1	AlphaFold prediction	1–735
DGKβ	Q9Y6T7	AF‐Q9Y6T7‐F1	AlphaFold prediction	1–804
DGKγ	P49619	AF‐P49619‐F1	AlphaFold prediction	1–791
DGKδ	Q16760	1R79	NMR spectroscopy	216–286
		3BQ7	X‐ray crystallography	1141–1208
		AF‐Q16760‐F1	AlphaFold prediction	1–1214
DGKη	Q86XP1	AF‐Q86XP1‐F1	AlphaFold prediction	1–1220
DGKκ	Q5KSL6	AF‐Q5KSL6‐F1	AlphaFold prediction	1–1271
DGKε	P52429	AF‐P52429‐F1	AlphaFold prediction	1–567
DGKζ	Q13574	5ELQ	X‐ray crystallography	921–928
		AF‐Q13574‐F1	AlphaFold prediction	1–928
DGKι	O75912	AF‐O75912‐F1	AlphaFold prediction	1–1065
DGKθ	P52824	AF‐P52824‐F1	AlphaFold prediction	1–942

Abbreviation: NMR, nuclear magnetic resonance.

The region containing C1 domains is quite similar between DGKs and PKCs, but unlike in PKC, it is unclear whether it can bind to phorbol esters (DAG analogues). Some groups hypothesize that DAG binding occurs in the C1 domains, but conflicting data suggests caution as in many isoforms those C1 domains are atypical as key residues are absent and phorbol esters binding is reported only for DGKβ and γ C1 [18]. Ware et al. otherwise suggested that the C1 atypical domain, that could be found in all the DGKs except for the β and γ isoforms, influences DAG preferences [15, 19].

The structure of the DGK catalytic domain in eukaryotes has not yet been resolved. However, progress has been made in understanding the ATP‐binding site through quantitative LC‐MS, which identified the localization of ATP analogues on mammalian DGKs. These studies suggest the existence of an ATP‐binding cleft formed by both the catalytic and catalytic accessory domains [20].

Further insights can be gained by two soluble bacterial enzymes with structural homology to mammalian DGKs: YEGS of *S. typhimurium*/*E. coli* and DgkB from *S. aureus*.

YegS is structurally homologous to sphingosine and diacylglycerol kinases, featuring a bipartite catalytic domain with an N‐terminal α/β domain preceding a C‐terminal two‐layer β‐sandwich domain. ATP binding occurs in the cleft between the two domains [21]. In *E. coli* is endowed with phosphatidylglycerol kinase activity and its catalytic sites resembles NAD kinase active sites suggesting conformational rearrangements to allow interaction with membrane lipids [22].

DgkB from *S. aureus* has DGK activity and a similar organization with a bipartite catalytic domain and ATP binding in the cleft. Despite low overall sequence homology with mammalian DGKs it is possible to define conserved secondary structures and key catalytic residues [17].

This structural organization is putatively conserved in mammalian DGKs, indeed the catalytic domain is bipartite with the “accessory” and “catalytic” domains of all isoforms showing a high degree of structural and sequence homology, while the C1 domains exhibit less homologies [15]. Aside from the limited knowledge about the substrate binding site, there is little information about their conformational changes and the dynamics of catalysis [9] and furthermore, for several isoenzymes have been predicted and sometimes well characterized splicing alternatives with distinct features [23, 24, 25, 26, 27]. This lack of detailed structural information explains why, at present, the development of DGK isoform‐specific modulators is quite limited.

Furthermore, it is believed that the structure and domains determine DGK's function but also cellular localization [11, 28].

### 2.1 Type I DGKs

#### 2.1.1 DGKα

DGKα is the first discovered and most studied isoform, as research on its role in both physiological and pathological processes has highlighted its significant impact. For this reason, several efforts have been made to solve its 3D structure to understand its function and to develop potential selective inhibitors. The DGKα structure is characterized by the presence of a recoverin homology domain (RVH), two EF‐hand motifs, two C1 domains, and a kinase domain. RVH and EF motifs in particular are key regulators of DGKα activity, because they maintain DGKα in an inactive state interacting with the C1‐catalityc region, but the EF motif binding with Ca^2+^ ions allows a conformational change that let the activation of this isoform [29, 30] and regulates its translocation to the membrane [31]. Recently, it was also discovered that the RVH mediates the interaction with the Wiskott‐Aldrich syndrome protein, which inhibits the activity of DGKα [32]. Other reported interactions verify for instance between DGKα and tyrosine‐protein kinase Src (SRC) through SRC's SH3 domain and the proline‐rich sequence at the C‐terminal of DGKα [33], as well as between and focal adhesion kinase 1 (FAK) protein's FREM domain and DGKα's catalytic domain [34]. A direct interaction may also occur between DGKα and the Src kinase family member Lck [35]. In addition to the structure predicted by AlphaFold, further insights into the N‐terminus of human DGKα have been obtained through NMR spectroscopy [10]. In addition, Takahashi et al. reported the X‐ray crystallography structure of human DGKα EF‐hand domains bound to Ca^2+^ [11]. Specifically, this study aimed to monitor calcium binding to EF domains and the conformational changes induced, postulating that, upon the Ca^2+^ binding, this isoform is subjected to a conformational change that triggers enzymatic activity. Apart from calcium ions, phosphatidylserine (PS) is another important actuator, because its binding to the catalytic domain promotes enzyme activation. Additionally, several phosphoinositide 3‐kinase lipid products and sphingosine can activate DGKα [36]. Specific phosphorylations have also been shown to trigger its activation and translocation across different cell compartments [31]. An example is the translocation of DGKα from the nucleus to the cytoplasm following serum stimulation after a period of starvation, where the enzyme shifts from cytoplasm to the nucleus. This nuclear export indeed requires the phosphorylation of DGKα at Tyr‐218 by the tyrosine kinase c‐Abl, in turn phosphorylated by c‐Src [37]. Interestingly, cell stimulation with growth factors or the TCR triggers Src family activity toward Tyr‐Y335 of DGKα promoting its activation and membrane recruitment [33, 35]. Thus DGKα localization is strictly regulated by extracellular signals. Putatively also DGKα own activity influences localization as it is required for membrane translocation in some systems [38] while in others inhibiting the catalytic activity prolongs membrane residence [39].

The Ensembl genome browser [40] reports that the DGKα‐human gene DGKA (ENSG00000065357) can generate 49 different transcripts, of which 16 contain an open reading frame (ORF). Indeed, several splicing variants of the canonical 735 amino acids ‐80 kDa form have been predicted, and their presence occasionally associated with pathology such as the case of a catalytically inactive variant causing juvenile periodontitis [27].

According to the Human Protein Atlas [41] (proteinatlas.org, version 24.0) and the literature [42, 43], DGKα is highly present in several tissues, in particular in lymphoid tissues such as spleen and tonsils. Focusing on the RNA single cell type specificity it is highly abundant in particular in T cells and in squamous epithelial cells among others.

DGKα can be found in different cell compartments depending on the cell type and the specific pathway involved. It is mostly located between the cytosol and the membrane, where it regulates, for example, T cells activation, lipid metabolism and signaling, as well as multivesicular bodies maturation [44]; it could also stay in the nucleus, where it regulates cell cycle progression [45] and proliferation [46].

#### 2.1.2 DGKβ

This 804 amino acids protein comprises again a recoverin homology domain, two EF‐hand domains, two C1 domains and a kinase domain according to UniProt and the literature, for a molecular weight of 90 kDa [47]. The structure of human DGKβ has been entirely predicted only by AlphaFold.

This isoform, together with α, and γ, possesses a EF‐hand domain. However, it was observed that the isoform‐specific EF‐structures differ for Ca^2+^ affinity and related conformational changes [30]. Although it is unclear if the C1 domains interact with DAG in all DGK isoform, it is reported that in the case of DGKβ and DGKγ they are able to bind DAG analogues [48].

According to Ensembl, up to 13 splicing alternatives could potentially be generated from the human gene (ENSG00000136267), but only six are predicted to code for a protein, and additionally few of them have been characterized [25]. For instance, comparing the “standard” DGKβ and a truncated splicing alternative (lacking part of the last exon, but still kinetically active), Caricasole et al. observed their different abundance in the diverse tissues, as well as their variable subcellular localization, being one variant at the plasma membrane, whereas the other one mainly localized within the cytoplasm [49].

DGKβ is particularly abundant in the brain, including regions as the striatum and the hippocampus [50]. In the Human Protein Atlas is additionally reported a messenger RNA (mRNA) enrichment in excitatory and inhibitory neurons, and oligodendrocyte precursor cells. DGKβ may regulate the actin filament assembly in neurons, processing, for example, the spinogenesis [51]. Additionally, changes in its localization or the catalytic activity could influence emotional and cognitive behavior, being DGKβ enriched in brain area controlling these functions [51]. In general, DGKβ is localized in the cytoskeleton and at the perisynaptic membrane [52]. In the study of Kobayashi et al. for instance, GFP‐DGKβ was distributed in the cytoplasm of COS7 cells, associated with the cytoskeleton, in particular the actin filaments [53].

#### 2.1.3 DGKγ

The full‐length human enzyme is constituted by 791 amino acid residues with a total molecular weight of approximately 89 kDa. According to the Ensembl database (ENSG00000058866), there are 11 splicing variants of which at least 4 protein coding. In this context, an inactive splicing version, without 25 amino acids in the catalytic region, is reported in the literature [25].

Also in this case, there are not experimental evidence of the protein structure, aside from the predictions performed by AlphaFold.

It is reported that DGKγ is abundantly expressed in the brain [54] and in the retina, although its role there is quite unexplored [55]. According to Human Protein Atlas, a low level of DGKγ protein can be found in other tissues, while RNA single cell type specificity indicates oligodendrocyte precursor cells, astrocytes, excitatory and inhibitory neurons as cell type enhanced. Very recently Zhang et al. demonstrated that DGKγ gene is abundantly expressed in hepatocarcinoma vascular endothelial cells, where it promotes tumor angiogenesis and immune‐evasion [56].

Using GFP‐DGKγ, Matsubara et al. observed a progressive translocation from the cytoplasm to the nucleus. This translocation is independent of kinase activity but depends on the presence of C1 domains. Putatively, nuclear DGKγ may be involved in cell‐cycle regulation [57]. DGKγ has been found also in the Golgi of rat aortic endothelial cells [58] and adrenal cells [59].

### 2.2 Type II DGKs

#### 2.2.1 DGKδ

Type II DGKs have two C1 domains and a kinase domain, similar to type I DGKs. However, in type II DGKs the two portions of the kinase domain are well separated. Additionally, these DGKs contain a pleckstrin homology (PH) domain [25], which mediates binding with phosphatidylinositol 4,5‐bisphosphate particularly in the δ and the η isoforms [60]. They also have a sterile α motif (SAM domain) that facilitates protein‐protein interaction and oligomerization. There is partial structural information of this isoform as Harada et al. were able to crystallize and reconstruct the structure of the DGKδ SAM domain (namely the 1141–1208 amino acids portion), further exploring its polymerization as a regulator of the enzyme localization. This domain appears to prevent translocation from the cytosol to the plasma membrane by mediating the formation of DGKδ polymers [13]. Moreover, the NMR reconstruction of the 216–286 portion, actually the C1 domain, in complex with two Zn^2+^ ions have also been deposited in PDB [12].

Observing the protein expression scores in the Human Protein Atlas it is evident that DGKδ is extremely ubiquitous, being present in several different cells and organs. As shown in the Ensembl database, the gene coding for DGKδ (ENSG00000077044) can originate 14 transcripts, with at least 5 of them potentially coding for a protein. One of the characterized splicing alternatives has also a Pro‐rich and Glu/Asp‐rich sequence at the N‐terminal and with an increased length (1214 instead of 1170 amino acids) and weight (135 instead of 130 kDa) [23]. The in‐depth characterization performed by Sakane et al. highlighted the profound differences between the “original” DGKδ1, and the second splicing alternatives, DGKδ2, showing also different tissue expression patterns: DGKδ2 transcripts have been found in several healthy and tumor tissues, while the DGKδ1 ones were detected in a few compartments, that is, in the ovary and the spleen and certain cancer cells. Moreover, their subcellular compartmentalization differs with DGKδ1 selectively recruited to the plasma membrane by phorbol esters in a PH‐dependent manner [23]. In another study, it was demonstrated that DGKδ associates to the endoplasmic reticulum (ER) through the SAM domain and inhibits the ER‐to‐Golgi anterograde transport through both the SAM and the PH domains [61].

#### 2.2.2 DGKη

DGKη gene (DGKH, ENSG00000102780) has 10 predicted splicing alternatives, 6 of which at least coding for a protein. The full‐length protein (DGKη2) weights approximately 135 kDa for 1220 amino acids and presents a SAM domain and a potential PDZ‐binding domain. As in the case of DGKδ, the SAM domain mediates oligomerization and subcellular localization. This makes DGKη2 more similar to DGKδ rather to the “first” DGKη discovered (DGKη1) that does not present this particular domain [26]. Other splicing alternatives have been characterized, namely DGKη3, that lacks both SAM domain and exon 26, and DGKη4, which has the kinase domain truncated [62]. DGKη enzymes are expressed in various tissues, with particularly high levels in the brain. They are also found in endocrine tissues, the testis and the gastrointestinal tract, indicating their ubiquitous presence in the human body. Furthermore, the in‐depth characterizations conducted by Murakami et al. on the splicing alternatives revealed that these variants apparently possess a different tissue and subcellular localization, as well as they could be involved in different processes, because they behave differently in response to the same stimulus. For example, under osmotic stress, DGKη1 translocates from cytoplasm to punctate vesicles where it colocalizes with endosomes in COS‐7 cells. Upon removal of the stimulus, it rapidly redistributes throughout in the cytoplasm, whereas η2 remains localized in the vesicles [24]. In another model, NEC8 cells, upon the osmotic stress stimulation DGKη3 instead was partly translocated to the plasma membrane [24, 62].

#### 2.2.3 DGKκ

Discovered in 2005, this isoform is the most recent addition to the Type II DGKs, sharing structural similarities with δ and η. However, it also exhibits unique structural and functional properties. First of all, it presents a Pro‐rich region, 33 tandem of glu‐pro‐ala‐pro (EPAP) repeats, and Ser‐Pro repeats at the N terminus. Besides, it lacks the SAM domain, therefore DGK*κ* is unable to form homo‐oligomer structures, but presents a PDZ binding domain, which mediates the interaction with PDZ domain present on different proteins [63]. Also its subcellular localization is quite different, because it stays at the cell periphery, in particular at the plasma membrane through its C‐terminal portion, even in absence of a stimulation, and it is inhibited by Src‐mediated phosphorylation, suggesting specific regulatory mechanisms [64].

The predicted structure of DGKκ consists of 1271 amino acids, resulting in an approximately 142 kDa protein. Interestingly, only one transcript is reported in Ensembl for its gene (ENSG00000274588).

Imai et al. observed that DGKκ mRNA and protein expression is highly restricted, being primarily found in the testis [64]. Otherwise, RNA single cell type specificity in the Human Protein Atlas indicates its expression in NK‐cells, late spermatids, oligodendrocyte precursor cells, inhibitory and excitatory neurons.

### 2.3 Type III DGKs

#### 2.3.1 DGKε

DGKε is the sole member of Type III DGKs due to distinct properties and features. DGKε is the smallest known DGK, consisting of 567 amino acids with a molecular weight of approximately 64 kDa. According to Ensembl, its gene (ENSG00000153933) has 6 splicing alternatives, 4 of which contain an ORF although their biological significance is still unknown.

The structure is quite simpler compared to other family members: it begins with an hydrophobic segment (probably forming a bitopic transmembrane helix or as monotopic re‐entrant helix [65]), followed by two C1 domains and the kinase domain. The transmembrane helix appears to anchor DGKε to specific membranes, such as the plasma membrane or the endoplasmic reticulum [65, 66]. DGKε also contains a lipoxygenase (LOX)‐like motif in the catalytic accessory domain [67]. Noteworthy, several mutations in this site have been shown to modify enzyme activity, probably perturbing lipid binding site, changing its peculiar substrate specificity [65]. Indeed, this isoform is also quite unique between the other DGKs because of its preference for a particular species of DAG: 1‐stearoyl‐2‐arachidonoyl glycerol (SAG), a lipid intermediate of the PI‐cycle. It seems that the LOX motif is involved in SAG binding, and furthermore the lipid composition and biophysical properties of the membrane containing SAG influence DGKε substrate specificity [68].

DGKε is present in several tissues and organs, including the endothelium, and in cells as platelets and podocytes [69]. As other isoforms, also this one is quite abundant in the brain, especially in the cerebral cortex, the hippocampus, and the cerebellum [70], as well as in the testis, according to the Human Protein Atlas.

### 2.4 Type IV DGKs

#### 2.4.1 DGKζ

DGKζ is the first identified member of the group IV DGKs and, along with DGKα, is among the most extensively studied isoforms. The full‐length product consists of a 928 amino acids protein, with a molecular weight of 104 kDa. At least two splicing alternatives are known [48] but, according to the Ensembl database, its gene (ENSG00000149091) has 24 different transcripts, of which 11 possess an ORF.

Although a small portion of the protein (from amino acid 921 to 928) in complex with SNX27 PDZ has been analyzed through X‐rays crystallography [14], the entire structure remains to be fully explored and it has been predicted by AlphaFold nowadays. Saito et al. were able to purify the full‐length human enzyme through a baculovirus‐insect cell expression system but, to date, there is not addition information about its 3D structure [71].

DGKζ structure comprises two C1 domains and a catalytic region, as all the DGKs. Its peculiarity lies in the presence of four ankyrin repeats followed by a PDZ‐binding domain after the kinase domain, as well as a myristoylated alanine‐rich protein kinase C substrate (MARKS) domain between the C1 and the catalytic region, which is homologous to the phosphorylation‐site domain of the MARKS protein.

DGKζ is able to migrate from the nucleus to the cytoplasm and vice‐versa [72] thanks to a nuclear localization signal partially located in the MARKS domain and the nuclear export signal located in the catalytic domain [73]. Phosphorylation of the MARKS domain is essential for the translocation from the cytosol to the plasma membrane in response to certain stimuli, for example, upon G protein‐coupled receptors stimulation in T lymphocytes [74]. On the other hand, the PDZ‐binding domain facilitates interactions with other proteins, as previously mentioned.

This isoform, like others, is ubiquitously present in the human body [52]. It is highly expressed in the brain and is also enriched in various organs, such as bone marrow, lymphoid tissues, and the respiratory system. Looking at the RNA single cell type specificity reported in the Human Protein Atlas, extravillous trophoblasts, NK‐cells and excitatory neurons present an enhanced expression.

#### 2.4.2 DGKι

DGKι is the second member of the group IV DGKs. It was discovered and characterized by Ding et al. [75], who identified this new isoform in human retina and brain libraries. Like DGKζ, it has two C1 domains, a MARKS homology domain, a catalytic region, four ankirin repeats and a PDZ binding domain. Its gene (ENSG00000157680) possesses 14 splicing variants, 6 of them containing an open reading frame. Some splicing alternatives, deprived of the ankirin repeats one lacking a part of the catalytic domain, have actually been characterized in rat brain tissue [76]. The full‐length protein weights 117 kDa and is composed of 1065 amino acids.

Despite its high structure similarity to DGKζ and a similar localization inside the nucleus or in the cytoplasm [72], it regulates cellular pathways differently. For example, DGKζ reduces Ras signaling by decreasing RasGRP1 activity, while DGKι enhances Ras signaling [77], suggesting isoform specific binding partners.

Regarding the protein tissue distribution, there is very few information about in the Human Protein Atlas, where it is reported that the choroid plexus, the retina and the thyroid gland are RNA tissue enhanced, while the RNA single cell type specificity reveals excitatory and inhibitory neurons and oligodendrocyte precursor cells as groups enriched. Literature reported its localization in brain and retina [75].

### 2.5 Type V DGKs

#### 2.5.1 DGKθ

This isoform belongs to the group V DGKs. It differs from the other DGKs because it possesses three C1 domains, whose capability to bind DAG is still under debate as previously mentioned. It also contains a Ras‐association (RA) domain within the pleckstrin homology domain that may not interact with Ras [78]. In addition, it has a proline/glycine‐rich region near its N‐terminal, whose function has been mostly postulated, because it possesses pXPXXP motif, typically found on SH3 domain‐binding sites [78, 79]. Some splicing variants are reported but not characterized: a total of 5 (4 with an ORF) according to Ensembl (ENSG00000145214). The full‐length product is composed of 942 amino acids for a 101 kDa protein. The structure is entirely predicted, although attempts to purify sufficient amounts of the protein for in‐depth structural characterization are ongoing [9].

DGKθ translocates from the cytoplasm to the nucleus [52]. It can also move from the cytosol to the plasma membrane in response to the activation of G protein‐coupled receptors and protein kinase C [80]. Furthermore, DGKθ catalytic activity is inhibited by the binding with activated RhoA [81].

DGKθ is expressed in various tissues, especially in the brain, but also in the kidney, intestine, liver, and arteries [82]. It has been found in nuclear speckles in several cell lines [83]. Its role in the regulation of the neurotransmission has been extensively studied, establishing its key role in synaptic vesicle recycling [84].

## 3 DGKs' Role in Physiological Processes

DAG and PA are lipid molecules fundamental for at least three biological processes, namely lipid metabolism, control of membrane curvature, and recruitment of selected proteins to specific membrane domains. The role of DGKε in enriching the phosphoinositide pool with polyunsaturated fatty acids at glycerol position 2 exemplifies the involvement of DGKs in lipid metabolism, a function clearly demonstrated in the brain [85], where many DGKs are highly expressed. Lipid metabolism is also closely related to membrane shape. Both DAG and PA have small headgroups relative to their two fatty acid tails, which support negatively curved membranes. However, PA differs by having a negatively charged headgroup. Indeed, DAG has a structural role in establishing membrane curvature and organelle morphology which can be manipulated by DGK activity [86]. Similarly, PA accumulates at areas of membrane curvature on the inner, negatively curved leaflet [87]. Finally, membrane dynamics and signaling result from a complex interplay between lipids and proteins. This is evidenced by the role of DAG and PA as second messengers, which regulate various pathways by targeting an ever‐growing number of protein to specific subcellular sites [6]. Usually, multiple lipid species are required to localize and activate a protein into a membrane domain as evidenced by the synergistic recruitment of the PX domain of p47phox by phosphatidylinositol 3,4‐bisphosphate and PA [88] or conventional PKC binding to DAG and anionic phospholipids [89].

Each DGK family member exhibits a specific expression pattern, and different isoforms can coexist within the same cell, indicating their nonredundant roles in various physiological processes. Conversely, an “incorrect” DGK activity was associated with the development of diverse pathologies or indicated to sustain mechanisms that promote the pathology itself. Thus, DGKs, by balancing or unbalancing DAG and PA levels, are involved in both physiological and pathological mechanisms.

**Figure 2:**
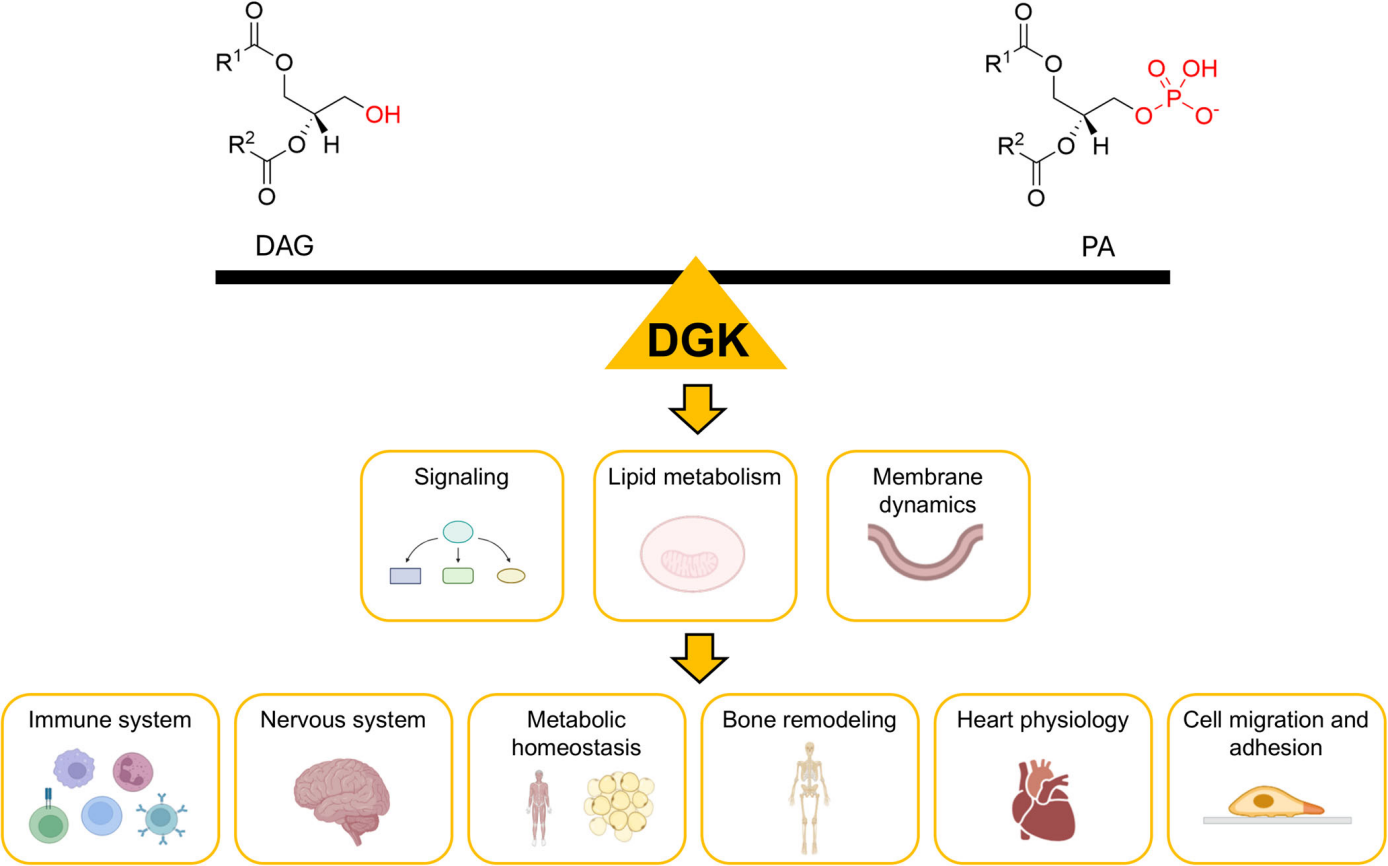
Summary of DGK role in cell and organs physiology. Controlling the DAG/PA balance, DGKs modulate different biological processes: signaling, lipid metabolism, and membrane dynamics. These in turn regulates several biological functions. Created with BioRender (https://www.biorender.com/). [Color figure can be viewed at wileyonlinelibrary.com]

It is extremely difficult to summarize all the roles that DGKs play in cell and organ physiology that make them attractive targets for drug development [90]. To our knowledge, the most explored DGK's function are immune response modulation, involvement in the central nervous system function and brain development, metabolic homeostasis, bone remodeling, heart physiology, cell adhesion and migration, here briefly summarized (Figure 2). For specific topics, readers can refer to excellent reviews focusing on DGKs and immunity [91], DGKs in neurons [92], DGKs and metabolic syndromes [93].

**Figure 3:**
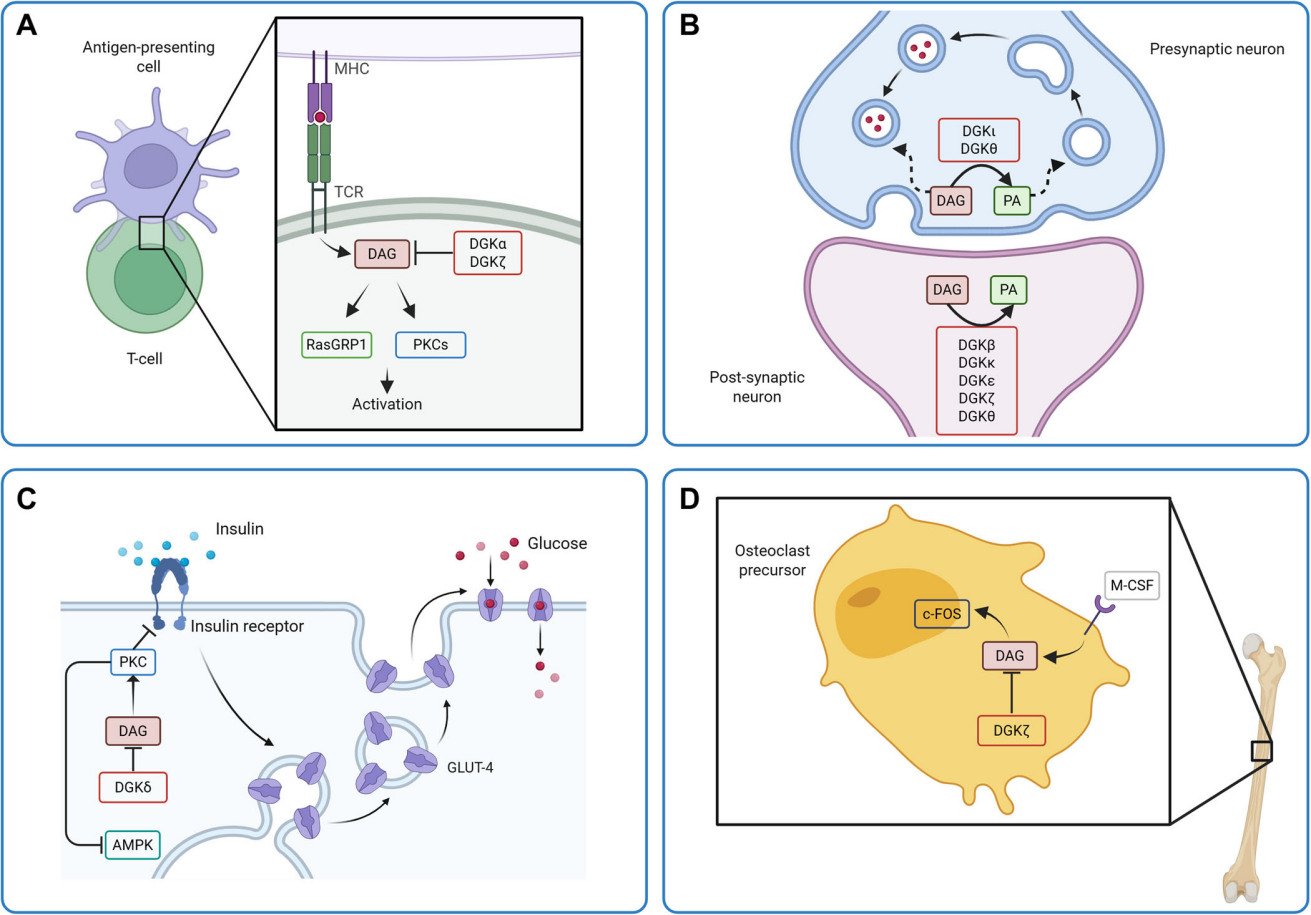
Some roles of DGK in physiological processes. (A) Immune responses. (B) Pre‐ and post‐synaptic processes. (C) Glucose homeostasis. (D) Bone remodeling. Created with BioRender (https://www.biorender.com/). [Color figure can be viewed at wileyonlinelibrary.com]

First of all, DGKs have a crucial role in the regulation of immune responses [94] (Figure 3A). Briefly, when T lymphocytes contact a non‐self antigen presented by antigen‐presenting cells, T cell receptor (TCR) triggers specific pathways, leading to cell expansion, differentiation, and effector responses which are modulated by coreceptors and cytokines. In this context, activating and inhibiting signals work together to modulate T cell behavior and prevent incorrect or uncontrolled responses. DAG and calcium are the key TCR second messengers, sufficient to trigger T cell activation in vitro. At the immune synapse indeed, DAG is necessary for both MAPK pathway activation through the recruitment of RasGRP1 and also regulates PKCs activity together with calcium leading to IL‐2 production. Therefore, DGKs metabolize DAG and act as negative regulators of T cell activation, while DGK‐mediated PA production is required for cytokine‐induced cell proliferation. The DGKζ isoform plays a quantitatively major role in this process, while DGKα acts at the periphery of the immune synapse to localize the DAG signaling [95, 96, 97, 98]. Fine‐tuning DGKα activity is crucial because strong TCR activation rapidly reduces its activity via a signaling pathway involving the SAP adaptor and the Wiskott‐Aldrich protein. TCR stimulation also decreases DGKα expression in a FoxO‐dependent manner [32, 99, 100]. DGKs are also important in thymus, where the α and ζ isoforms are necessary for the development of T cells and type I natural killer T cells (iNKT). For instance, DGKα and DGKζ appear to cooperate to establish iNKT cells development and homeostasis controlling DAG‐mediated activation of the PKCθ‐IKK‐NFκB and the RasGRP1‐Ras‐Erk1/2 pathways. Their double silencing indeed resulted in severe developmental defects, while the silencing of a single isoform (α or ζ) did not alter significantly this process [101]. Moreover, their absence also reduced the number of CD4+ and CD8+ single‐positive thymocytes, impairing positive selection, which was partially compensated by PA administration, suggesting that not only DAG metabolism but also PA production is fundamental for the outcome of this event [102]. In addition, DGKζ is also important in regulating the immune synapse in B cells [103]. Furthermore, less explored but equally important is the role played by DGKs in leukocytes and in general in the innate immunity, only partially elucidated. For example, DGKα in neutrophils is essential for limiting oxidative burst and promoting migration [104], regulating cell adhesion and other functions by fine‐tuning PA and DAG levels. Instead, DGKγ is crucial for mast cells degranulation through the control of Ca^2+^ ions influxes [105].

As previously mentioned, several DGK isoforms are highly expressed in the brain in line with a major role of PA and DAG in different nervous system functions, such as the myelinization and neurotransmission. Indeed oligodendrocytes' DGKα for example may be involved in myelin production and regulation [55], but also other isoforms, as the β one, play crucial roles in the brain [47, 55]. DGKθ for instance seems involved in the modulation of synaptic vesicles recycling [106], as DGKι also resulted to be crucial at the presynaptic level by controlling DAG and its effectors, as Munc‐13, which plays a role in synaptic vesicle priming [107], while other DGKs are more involved in post synaptic processes [55, 84, 92] (Figure 3B). Some DGKs are also involved in brain development, such as DGKθ [108], and DGKδ [109]. Moreover, DGKε may participate in the modulation of neuronal signaling pathways linked to synaptic activity and neuronal plasticity by its involvement in the PI‐related lipid signaling [85].

The control of DAG levels is extremely important also for glucose homeostasis (Figure 3C). Normally indeed insulin signaling is blocked through the dissociation of insulin receptor substrate from the insulin receptor after its phosphorylation by PKC, in turn recruited by insulin itself. However, DAG can also recruit PKC, and therefore a decreased DGKδ activity can decrease insulin signaling by increasing DAG pool, therefore unbalancing PKC activation [110]. Conversely, DGKδ overexpression has several positive effects, because it decreases fat mass, promotes glucose tolerance and protects against obesity [111]. Furthermore, DGKδ haploinsufficiency results in an in impaired regulation of lipid oxidation and storage caused by a decreased AMP‐activated protein kinase (AMPK) signaling pathways [112, 113]. Also, DGKζ, ε, and θ play moreover crucial roles in glucose and energy homeostasis, as reviewed by Massart [93].

DAG‐related pathways are also involved in bone remodeling. Bone homeostasis is a delicate process finely tuned by the coordinated activity of various cells, as osteoblasts and osteoclasts and their respective precursors. DGKs may obviously participate in this complex scenario by regulating for instance osteoclastogenesis, being the different isoenzymes expressed by these cells [114]. For example, M‐CSF, also present in different pathologies such as rheumatoid arthritis, induces the generation of DAG, which is necessary for c‐Fos expression, that in turn is a transcription factor involved in osteoclast differentiation. DGKs obviously are involved in this process (Figure 3D), and in case of DGKζ deficiency, DAG accumulates and the osteoclastogenesis increases due to c‐Fos rise [115]. Moreover, DGKζ is involved in osteoclast differentiation and bone resorption under inflammatory conditions. The level of this isoform is indeed lowered during differentiation and/or inflammation, probably due to its proteolytic degradation. In this context, the NF‐κB pathway is potentiated in response to TNF‐α stimulation in pathological conditions, and results in an increased expression of RANKL, which in turn promotes osteoclast activity and bone resorption [114].

Only partially explored is the role of DGKs in heart physiology and protection from related diseases. Different isoforms, such as α, ε, and particularly ζ, are involved in this context and show differential expression following heart‐related pathologies. It was indeed demonstrated the involvement of the Gαq‐phosphoinositide signaling and in particular of PKC in cardiac hypertrophy and heart failure, thus DGKs, by controlling DAG levels, are also participating to this scenario [116, 117]. Under chronic pressure overload for instance DGKε reversed cardiac dysfunction and improved survival in a mouse model by controlling DAG levels and transient receptor potential channel‐6 expression [118].

Finally, lipid gradients modeled by DGKs control cell migration and adhesion. Indeed in epithelial cells, DGKα‐produced PA recruits Rho‐GDI and atypical PKC that coordinate the complex signaling necessary for protrusion formation and directed cell migration together with RCP protein driving integrin recycling [119, 120, 121]. There are experimental evidence that also DGKζ and DGKγ can orchestrate similar processes in fibroblasts [122].

The crucial role of DGKs in biological processes is also evinced by the numerous studies performed in DGK‐deprived mice, summarized in Table 2. However, few studies in vivo were able to deal with the intrinsic redundancy in this family by knocking out multiple isoforms at the same time.

**Table 2:** Lessons from DGK‐deprived mice.

Isoform	Lessons from DGK deprived mice	References
DGKα	DGKαs play important roles in regulating T cell activation and anergy, macrophage activation and responses, T_H_ cell differentiation, and iNKT cell development	[101, 123, 124, 125]
DGKβ	DGKβ controls neurite spine formation and behaviour alterations	[126, 127]
DGKγ	DGKγ regulates motor coordination through the involvement in cerebellar long‐term depression and in the dendritic development of Purkinje cells	[128]
DGKδ	DGKδ is important in neuronal activity, where it regulates the extension of long axons/neurites. Moreover, it is necessary for a proper expression of EGFR protein and its haploinsufficiency alters glucose metabolism	[109, 129, 130, 131]
DGKη	DGKη absence is involved in mania‐like disorders, modulates gene expression in several pathways and is associated to a dopaminergic hyperfunction	[132, 133, 134]
DGKκ	No data available	—
DGKε	DGKε is involved in seizures, but also in glucose tolerance and lipid metabolism	[85, 135]
DGKζ	DGKζ plays important roles in regulating T cell activation and anergy, T_H_ cell differentiation, iNKT cell development, the regulation of actin polymerization, and LFA‐1–mediated adhesion at the B cell immune synapse. It is involved in protease‐mediated allergic airway inflammation and plays a significant role in regulating growth and metabolic processes.	[77, 101, 103, 123, 124, 136, 137, 138, 139]
DGKι	DGKι regulates RasGRP3 and Rap1, modulating Ras signaling. Its ablation reduces Ras‐induced tumor formation.	[77]
DGKθ	DGKθ is involved in SV recycling	[106]

## 4 Pathologies Associated with Deregulated DGK Activity

Since both DAG and PA are finely regulated in complex scenarios involving multiple cell types, imbalances caused by their accumulation, resulting from decreased or increased DGK activity, can lead to severe consequences. In Table 3 are listed some examples of pathologies correlated with a absent/reduced or a normal/increased activity of DGK isoforms.

**Table 3:** List of pathologies associated with DGKs.

Isoform	Absent/reduced activity	Normal/increased activity
DGKα	Localized aggressive periodontitis [27]	X‐linked lymphoproliferative disease[140]^(p1)^ [141], Wiskott‐Aldrich syndrome [32], allergic inflammation and airway hyperresponsiveness of asthmatic airways [142], cancer [143, 144, 145, 146]
DGKβ	Neurological disorders [126]	—
DGKγ	—	Cancer [56]
DGKδ	Neurological disorders [109], obesity, and type 2 diabetes [113]	—
DGKκ	Neurological disorders [147]	—
DGKη	Neurological disorders [132, 133]	Cancer [143]
DGKε	Obesity and insulin resistance [148], atypical haemolytic uraemic [69]	Huntington's disease [149]
DGKζ	Osteolytic bone destruction [114]	Allergic inflammation and airway hyperresponsiveness of asthmatic airways [142, 150], cancer [143, 151, 152, 153]
DGKι	—	Cancer [154]
DGKθ	—	—

An example is localized aggressive periodontitis (LAP), an inflammatory syndrome characterized by periodontal bone loss. It has been demonstrated that polymorphonuclear neutrophils in LAP show increased DAG accumulation caused by a truncated DGKα protein (lacking exon 10). This truncated protein acts as a dominant‐negative transcript against the full‐length enzyme [27]. In this case thus, enzyme activity restoring could be beneficial for the patients.

As DGKs have several crucial roles in the nervous system, their dysregulation is associated with neuronal diseases. Association studies correlated several polymorphisms located in DGKH with bipolar disorder [155, 156], and furthermore the A allele of the A/G DGKH polymorphism is associated to an increased volume of amygdala, probably modulating its activity influencing some characteristic premorbid personality traits of this pathology [157]. Variations at the DGKH locus have also been correlated with unipolar depression and adult attention‐deficit/hyperactivity disorder [158]. Other investigations found variations correlated to Parkinson's disease in the cyclin G‐associated kinase/DGKQ (DGKθ gene) region, suggesting a putative involvement of this isoform in this context [90]. Studies on DGKβ knockout mice evidenced how this isoform may be important for memory and emotions by regulating spine formation and branching for instance [47]. Moreover, abnormalities of DGKη or DGKκ have been correlated to neural disorders [47, 159] as well. DGKη‐deprived mice show a phenotype similar to mania of bipolar disorder [132, 133], while DGKκ instead seems to be involved in the Fragile X syndrome. This is indeed caused by the absence of the protein Fragile X Mental Retardation Protein (FMRP) in neurons, which results in a deregulation of DGKκ expression. The resulting DAG/PA imbalance correlates with dendritic spine abnormalities and impaired synaptic plasticity [147]. DGKδ absence instead relates to seizures in humans and mice [109]. DGKε is also related to neuronal disorders, as its ablation can for instance make neurons resistant to seizures [85], and it seems to contribute to Huntington's disease, because the negative modulation of DGKε activity prevented for example huntingtin protein activation of caspase‐3 [149].

In metabolic disorders, decreased DGK activity is present in type 2 diabetes and obesity. DGKδ, for instance, is constitutively expressed in insulin‐sensitive tissues and fundamental for adipocyte differentiation and maturation. Its expression is reduced in type II diabetes patients and its deficiency decreases AMPK signaling and lipid metabolism, most likely contributing to the pathogenesis of obesity and type 2 diabetes [113]. In diabetic nephropathy, one of diabetes' major complications, a possible therapeutic strategy is the activation of DGKα and DGKδ. DGKα, in particular, can reduce PKC activity in kidneys by decreasing DAG levels, while protecting podocytes (glomerular epithelial cells) through the PA production, preventing processes such as apoptosis [160]. On the contrary, DGKH emerged as biomarker for diabetic nephropathy in a recent bioinformatic investigation, but its role in this pathology remains largely unexplored [161]. Furthermore, it is well known that DAG species are not only important factors for cell signaling, but are also intermediates for phospholipids and triglyceride synthesis. In this context, DGKε appears to be particularly involved and appears to be crucial to control obesity and insulin resistance, where its deficient activity contributes to the pathology [148]. On the other hand, DGKζ role in muscles growth, insulin resistance, and obesity is multifaceted, with its participation in obesity physiopathology for instance [93].

Bone remodeling abnormalities can also be caused by DGK hypoactivity, as mentioned before. DGKζ for example appears to be a negative regulator of osteoclast differentiation and bone resorption, therefore limiting bone impairments under inflammation conditions. However, its absence could facilitate osteolytic bone destruction [114].

Atypical haemolytic uraemic syndrome was instead strictly associated to DGKε gene (DGKE) recessive mutations. This multifactorial pathology, part of the multifaceted group of the thrombotic microangiopathies, is characterized by different outcomes, such as nonimmune haemolytic anaemia, thrombocytopenia, and acute kidney injury. Differently from the typical haemolytic uraemic syndrome, that is generally caused by bacterial Shiga‐like toxins in children, the nonbacterial atypical form is associated with environmental factors and genetic alterations. In this context, various mutations have been found, such as loss‐of‐function mutations in complement factor H and complement factor I, as well as gain‐of‐function mutations in complement component 3 or, complement factor B [162]. Several analyses also linked DGKε activity loss with this syndrome, and furthermore different mutations seem to be associated with diverse clinical manifestations, as reported in the comparative analysis of the numerous case reports [69, 163, 164, 165]. In those patients the pathology seems complement‐independent, the arachidonic acid‐containing diacylglycerol signaling burst consequent to DGKE loss may induce platelet activation and thrombosis, decrease antithrombotic endothelial signaling [166] and induced actin cytoskeletal rearrangements in podocytes, but further studies are required to understand the exact mechanism [163] and tune the therapy accordingly [165].

Conversely, in some scenarios, the pathology is not caused by the absence or insufficient activity of DGKs but rather by their hyperactivity. This leads to PA accumulation, limits DAG‐related signaling, and disrupts the normal cellular environment. An example is when DGKs continue to phosphorylate DAG because the factors that normally regulate their action are not working properly, for instance are mutated. This phenomenon is typical of certain rare diseases characterized by immune response abnormalities. The first case is the X‐linked lymphoproliferative disease, a rare pathology characterized by the inability to counteract the Epstein‐Barr virus infection, resulting in an uncontrolled immune response that can't resolve the infection and may lead to hemophagocytic lymphohistiocytosis due to defective apoptosis of T cells. In addition, the patients, even those negative for the virus infection, are more prone to develop vasculitis, hypogammaglobulinemia, bone marrow hypoplasia, and malignant lymphoma. Mutations in the SH2D1A gene, which encodes for the signaling lymphocyte activation molecule–associated protein (SAP) causes type‐1 of this pathology. Present on T cells and NK cells, SAP is involved in the complex immune synapse signalosome upon the T cell activation, where it also promotes the inhibition of DGKα, and results for instance in the activation of MAPK pathway, cytokines expression, and NFκB activation. In the antigen‐experienced CD8+ cells, these events trigger the restimulation‐induced cell death program, promoting effector T cells clearance and preventing excessive lymphoproliferation. Therefore, when SAP‐driven DGKα inhibition fails, resistance to apoptosis and uncontrolled lymphoproliferation along with a weaker immune response to viral infection occur. Therefore, the administration of specific DGKα inhibitors can restore the restimulation‐induced cell death and may reduce the symptoms in murine models [140, 167].

Another example is the Wiskott‐Aldrich syndrome (WAS) case. This pathology is characterized by mutations of the gene that encodes the WAS protein (WASp), and is characterized by immunodeficiency, thrombocytopenia, and eczema among others, and an increased risk to develop autoimmune disorders and malignancies, such as lymphoma. WASp, present in non‐erythroid hematopoietic cells [168], is able to bind directly and inhibit DGKα upon TCR activation. The anormal DGKα activity in WAS patients may contribute to defective T cell signaling presence and the treatment with DGK‐specific inhibitors rescue at least cytokine induction in vitro [32]. Interestingly, DGKζ KO promotes glycoprotein VI expression in megakariocytes and platelet function [169], suggesting that DGK inhibitors may be useful also to counteract thrombocytopenia and bleeding.

DGKζ and DGKα also emerged as novel therapeutic targets for the treatment of allergic inflammation and airway hyperresponsiveness of asthmatic airways, being involved for instance in airway inflammation and airway smooth muscle cell contraction, and therefore their genetic ablation or pharmacological inhibition is under investigation to treat these pathologies [150]. In this case again DAG/PA‐related signaling pathways are involved in the adaptive immunity that causes the inflammation responsible for the allergic airway disease. DGKs have a multifaceted role in asthma pathology, because several cells are involved in this process, and they could be positively or negatively impacted. In brief, in the lung indeed this situation is orchestrated by a complex mechanism involving T helper 2 (Th2) CD4 + T cells and group 2 innate lymphoid cells, whose action recruits and triggers eosinophils, allergen‐specific B cells and in turn basophils, mast and goblet cells, resulting in and excessive airway smooth muscle contraction. At the end, this process culminates in damage of the lung parenchyma and the impairment of lung function. In this context, DGKs regulate several pathways in the different cells involved, improving or decreasing the asthma‐related factors [142].

One of the most active fields of research is DGKs' role in cancer, as the high expression level of these enzymes in several cancer types and their involvement in signaling make them attractive targets in a field that is giving the first results with phosphatidylinositol 3 kinase inhibitors, now in the market [170]. DGKα is probably the isoform most investigated in cancer, being overexpressed in several cancer subtypes, such as acute myeloid leukemia [143], melanoma [144], hepatocellular carcinoma [145], glioblastoma [146], and so forth. In melanoma cells for instance it helps to prevent apoptosis promoting NF‐κB activation via the PKCζ‐mediated Ser‐311 phosphorylation of p65/RelA [144, 171], while in hepatocellular carcinoma it sustains cell proliferation through the activation of the MAPK cascade [145], in non‐small cell lung cancer it promotes metastatic processes activating the SRC/FAK complex, modulating the WNT/β‐catenin and VEGF pathways [34]. In general, it is reported that stress conditions (as radiation therapy) enhance expression of DGKα in cells and its expression is particularly relevant in tumor‐infiltrating T cells [172, 173]. High DGKα activity on one side propels tumor cell growth and motility and on the other side makes tumor‐infiltrating T cells unresponsive to cancer cells, facilitating the immune‐escape. Indeed, DGKα is a major determinant of T cell anergy and together with enhanced DGKζ, makes tumor‐infiltrating CD8 and NK lymphocytes hyporesponsive by downmodulating DAG signaling to MAPK pathway. While the connection between immune‐checkpoint and DGK activity is still not clear, their inhibition robustly enhances immune responses against tumors [172, 174, 175]. Intriguingly, knockdown of DGKα and DGKζ also potentiates CAR‐T driven antitumor responses [176]. All of this makes DGKα/ζ inhibitors interesting for cancer immunotherapy as they offer a double effect: they potentiate immunesurveillance and decrease cancer cell growth.

By contrast, DGKβ seems to have a protective role against glioblastoma, as reduced expression of this isoform has been correlated with radioresistance in this specific pathology [177]. DGKγ instead plays a crucial role in the tumor microenvironment of hepatocellular carcinoma, because its increased expression, associated with the hypoxic conditions in tumor vascular endothelial cells, correlates with a poorer prognosis. In particular, DGKγ expression promotes angiogenesis and T‐reg differentiation, enhancing immune evasion. Intriguingly, this effect is not related to its kinase activity, but appears to be linked to its interaction and the stabilization of ZEB2, a key protein for epithelial‐mesenchymal transition [56]. Interestingly, DGKγ may have tumor‐suppressive properties in colorectal cancer [178] and is associated with a more favorable prognosis for acute myeloid leukemia [143], highlighting its tumor‐specific role. Higher DGKH expression has been associated with a shorter survival in acute myeloid leukemia, suggesting a possible involvement also of this isoform in the pathology [143] as well as DGKι‐coding gene (DGKI) in gastric cancer [154]. DGKζ is also involved in the cancer, being overexpressed in several tumors as acute myeloid leukemia [143], breast cancer [151], cervical cancer [152], and osteosarcoma [153]. Zhao et al. for example reported its involvement in the epithelial‐mesenchymal transition of triple‐negative breast cancer cells through the TGFβ‐SMAD signaling pathway [151]. Interestingly, DGKζ pays a major role in down modulating diacylglycerol signaling at the immune synapse [179]. Accordingly, DGKζ inhibition synergizes with checkpoint inhibitors in restoring tumor‐infiltrating lymphocytes activity toward cancer cells, similarly to what observed with DGKα and putting also this isoform at the center of intensive research for inhibitors [180, 181].

## 5 Modulating DGK's Activity

Given the wide range of physiological and pathological processes involving each DGK isoform, as well as the co‐expression of multiple isoforms in cells and tissues, it is clear that research into isoform‐specific DGK activators and inhibitors is essential. Such molecules are crucial for investigating the involvement of one or more isoenzymes in physiological or pathological mechanisms. Once the role of DGK activity in a pathology is established, it becomes possible to develop activators or inhibitors for targeted disease treatment.

Currently, there are few options for DGK activators, likely because antitumor research has focused more on DGK inhibition rather than activation. However, even with the focus on inhibition, most compounds developed so far target DGKα and/or DGKζ, largely overlooking the other isoforms. This gap, particularly concerning the less studied isoenzymes, is partly due to limited knowledge of DGK structure and an incomplete understanding of their individual features and functions. This unsolved problem also makes the design of structure‐based activators and inhibitors more challenging. Obtaining more information about their structure will most likely lead to an increase in research efforts in this area.

Indeed, the successes obtained with those two isoform demonstrate that, despite similarities in the catalytic domains, the design of isoform specific inhibitors is feasible.

### 5.1 Activators

The investigation into DGK activators is quite recent and largely unexplored at the moment, despite the association between DGK dysfunction and various pathologies. In addition, the few activators discovered often come from studies with the exact opposite aim, actually to find new inhibitors: while assessing the activity of various compounds, some unexpectedly displayed activation properties. The known activators are listed in Table 4.

**Table 4:** List of known activators.

Activator	Activated DGK Isoform	Key information	References
d‐α‐tocopherol 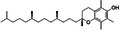	DGKα	Improves DGKα activity in a diabetic nephropathy mouse model at a dose of 40 mg/kg	[182]
KU‐8 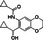	DGKα, DGKθ	Activates these two isoenzymes at 100 μM, but also inhibits DGKk and other isoforms	[183]
Fluoxetine 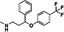	DGKα	Activates DGKα at 100 μM	[184]
phytochemical atractylenolide II (compound 419) 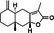	DGKθ	Activates DGKθ modulating sn‐1,2‐DAG‐PKCε signaling axis	[185]

One of the few studies in this area is the work performed by Hayashi and colleagues in the field of diabetic nephropathy. They found out that a d‐α‐tocopherol treatment attenuates the pathological conditions in a mouse model improving DGKα activity [182], after the demonstration that DGKα is translocated and activated by d‐α‐tocopherol and its derivatives in a previous study [186], encouraging a deeper investigation into other putative DGKα activators. From a random selection of 9600 molecules in the Core Library (Drug Discovery Initiative, University of Tokyo), they identified KU‐8 as a promising candidate. Treatment with 100 μM KU‐8 significantly enhanced kinase activity compared to the control and activated the enzyme in vitro. Surprisingly, this molecule also showed activating properties on DGKθ, but also inhibitory properties on other isoforms, especially DGKκ, as presented in the following section [183].

Other molecules able to potentiate DGKα activity emerged from the screening of Velnati et al. aiming to find new inhibitors. In particular, 100 μM of fluoxetine significatively improved DGKα activity in vitro. Also other compounds, actually pimozide, AMB1881676 or paliperidone, were able to improve the enzyme activity, even if not significantly at the dose considered. All these molecules have not been further characterized, even if a possible mechanism of action is postulated: they may improve the affinity for ATP or may allosterically activate the enzyme [184].

Zheng et al. identified a new DGKθ activator by screening an in‐house natural product library for molecules capable of inhibiting the sn‐1,2‐DAG‐PKCε signaling axis, which is involved in metabolic diseases related to obesity and insulin resistance. They indeed selected the phytochemical atractylenolide II (compound 419) for its success in modulating the target pathway, finding out later that its effects were due to its ability to allosterically activate DGKθ [185].

### 5.2 Inhibitors

As previously mentioned, significant efforts have been directed towards developing DGK inhibitors due to their potential as therapeutic targets. Consequently, there are more proposals focused on inhibition rather than activation. One potential approach is the use of broad‐spectrum inhibitors, which can downregulate the activity of all DGK family members. However, since different isoforms play distinct roles in physiology and pathology, most studies have focused on developing isoform‐specific inhibitors. The molecules proposed are listed in Table 5.

**Table 5:** List of DGK inhibitors.

Inhibitor	Inhibited DGK isoform	Key information	References
Dioctanoylethylene glycol 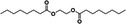	All DGKs	DAG analogue, Ki 58 μM	[187]
1‐Monooleoylglycerol 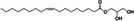	All DGKs	DAG analogue, Ki = 91 μM	[187]
Calphostin C 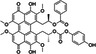	All DGKs	DAG‐competitive inhibitor, IC_50_ = 40 μM	[188]
Cochlioquinone A 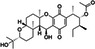	All DGKs	ATP‐competitive inhibitor. IC_50_ values for bovine thymus DGK at 2.3 μM, later reported a Ki = 3.1 μM	[189, 190]
Stemphone 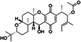	All DGKs	Probably ATP‐competitive inhibitor. IC_50_ values for bovine thymus DGK at 3.3 μM	[189, 191]
R59022 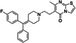	DGKα, DGKθ	More specific on DGKα and DGKθ. Used to study DGK's role in glucose transport, asthma, and other diseases. DGKα ED_50_ ∼25 μM	[167, 192, 193, 194, 195, 196, 197]
R59949 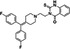	DGKα, DGKγ, DGKδ, DGKκ	Inhibits DGKγ, DGKδ, and DGKκ. Used to study DGKα role in lymphoproliferative diseases and XLP‐1 for instance. DGKα ED_50_ ∼18 μM	[167, 193, 194, 196, 198]
Ritanserin 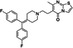	DGKα	Serotonin receptor antagonist with good pharmacokinetics properties. Studied for cancer treatment. IC_50_ value of 15 μM	[34, 199, 200]
JNJ‐3790339 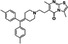	DGKα	Ritanserin derivative, more cytotoxic for cancer cells. IC_50_: 9.6 μM	[201]
AMB639752 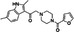	DGKα	Selectively inhibits DGKα (IC_50_ of 4.3 μM) while avoiding serotonin receptor off‐target effects	[184, 202]
CU‐3 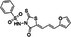	DGKα	Promotes apoptosis and IL‐2 production in T cells, efficiently reduces cancer cell viability. IC_50_. = 0.6 μM	[203, 204]
Compound A	DGKα, DGKβ, DGKγ	Inhibits DGKα, DGKβ, and DGKγ. IC_50_ for DGKα: 0.04 μM, for DGKβ: 0.02 μM, for DGKγ: 0.01 μM. Increases T cell response and cancer cell death.	[204]
DGKAI	DGKα, DGKβ, DGKγ	Effective in hepatocarcinoma models and enhances T cell immune responses in vivo. IC_50_ α: 0.01, β: 0.01, γ: < 0.01 μM	[205]
ISM4312A	DGKα	Specific to DGKα, shows antitumor activity in solid tumor models. IC_50_: 120 pM	[206]
Indole N‐methyl derivative 2	DGKγ	Inhibits DGKγ, IC_50_ value of 13 nM	[207]
KU‐8 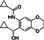	DGKκ, DGKι	Inhibits particularly DGKκ (80% of DGKk activity was inhibited by 10 μM of KU‐8), but also other DGKs are negatively affected by its addition	[183]
ASP1570	DGKζ	The treatment enhances both T and NK cell function, exploitable for antitumor purposes	[208, 209]
BAY2965501 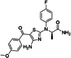	DGKζ	Selective DGKζ inhibitor, studied for solid tumors. Reported IC_50_ in CAS SciFinder 4.62 μM	[210, 211]
Momordicine I 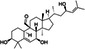	DGKζ	Not a direct inhibitor, reduce DGKζ mRNA and protein induced by the isoproterenol treatment.	[212]
BMS‐502 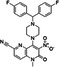	DGKα, DGKζ, DGKι	Enhances immune response against cancer. IC_50_ of 0.0046, 0.0021, and 0.0026 μM for DGKα, DGKζ, and DGKι, respectively	[213]
BMS‐332 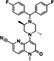	DGKα, DGKζ, DGKι	IC_50_ of 0.009, 0.008, and 0.01 μM for DGKα, DGKζ, and DGKι, respectively	[214]
U73122 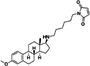	DGKθ	Inhibits DGKθ competing for the substrate (Ki ~20 μM)	[215]

A possible strategy to inhibit DGK enzymes, avoiding the focus on a single isoform, is the use of DAG analogues, such as dioctanoylethylene glycol (Ki = 58 μM) and 1‐monooleoylglycerol (Ki = 91 μM) [187]. Calphostin C, which can inhibit DGKs (half‐maximal inhibitory concentration IC_50_ of 40 μM), may also compete with DAG [188]. Other generic “DGK inhibitors” are for instance molecules able to compete with ATP, namely ATP‐competitive inhibitors. An example is Cochlioquinone A (IC_50_ values for bovine thymus DGK at 2.3 μM, later reported a Ki = 3.1 μM), extracted by Drechslera sacchari. Stemphone, extracted from the same source and structurally very close, probably possessing similar mechanism of action (IC_50_ values for bovine thymus DGK at 3.3 μM) [189, 190] was also used to treat experimental hypervascular contraction [191]. However, these generic inhibitors have seen limited experimental use, likely due to concerns about their poor isoform selectivity and potential off‐target effects.

Other DGK inhibitors are R59022 (6‐[2‐[4‐[(4‐fluorophenyl) phenylmethylene)‐1‐piperidinyl]ethyl]‐7‐methyl‐5H‐thiazolo[3,2‐alpha] pyrimidin‐5‐one) and R59949 (3‐[2‐[4‐[bis(4‐fluorophenyl)methylene]‐l‐piperidinyl] ethyl]‐2,3‐dihydro‐2‐thioxo‐4(lH)‐quinazolinone. They emerged from a deep screening where they showed an ability to inhibit DGK activity in human red blood cells membranes, platelets cells, and membranes [192, 198]. Studies on their selectivity and IC_50_ have produced conflicting results, largely due to variations in methodologies, which make comparisons challenging. To ameliorate DGK inhibitors search, Sato et al. developed a nonradioactive, single well, two‐step DGK assay system, and with this system they tested the selectivity of these two agents on all the DGK isoforms. The detailed analysis carried out demonstrated that these two molecules, even if quite similar from a chemical point of view, were able to inhibit different isoforms: between the Type I DGKs, α was quite affected by both and the ED_50_ measured were ∼25 and 18 μM for R59022 and R59949, respectively. Measuring the activity on the other isoforms in presence of 30 μmol/l R59022 or 20 μmol/l R59949, they found out that DGKγ's activity was significantly reduced (60%) only by R59949, while DGKβ's one was not influenced; among the Type II DGKs only δ and κ were partially inhibited by R59949 (20% and 30% reduction), while η activity was unvaried; on the other hand, DGKε was partially inhibited by R59022 (25% decreased activity), while Type IV DGKs were untouched by both agents; finally the θ isoenzyme was sensitive again exclusively to R59022 (25% decreased activity) [193]. Both these molecules represent one of the most popular proposals for DGK inhibition, and therefore there are several papers where they are employed to investigate DGK activity role in a peculiar phenomenon. Batista et al. used both molecules to study the influence of DGKs on HL‐60 cell differentiation, as DAG‐triggered PKCs accelerate this process. The observed differences were attributed to the distinct isoform specificities of the inhibitors [194]. Another example is the study of Hernandez‐Lara, where R59022 has been used also as DGK inhibitor to understand the mechanism for attenuating airway smooth muscle cell proliferation, responsible of asthma pathology. DAG increase through DGK inhibition probably induced PGE2 secretion via a PKC‐ERK1/2‐COXII axis. Secreted PGE2 potentiated PKA‐mediated anti‐mitogenic signaling by activating Gs‐coupled GPCRs [195]. Otherwise, they can be employed with the purpose to directly hit DGKα activity. Ruffo et al. for instance tested both inhibitors to pharmacologically downregulate DGKα in a X‐linked lymphoproliferative disease model to understand how its uncontrolled action, not limited as usual by SAP protein, here absent or not properly working due to a genetic alteration, is related to the development of the disease. They confirmed their hypothesis: the absence of SAP permits DGK activity, leading to decreased DAG levels and impaired restimulation‐induced cell death in lymphocytes. Treatment with the inhibitors successfully reversed this pathological phenotype [167]. A very intriguing situation can emerge when the results between pharmacological and genetic ablation are different: it is, for example, the case presented by Arranz‐Nicolás et al. [216]. They compared the effects of commercial DGK inhibitors, namely R59022, R59949, and ritanserin (presented below), on T cell responses with those achieved by DGKα and DGKζ silencing. The results were quite different, as DGKα inhibition is required for strong T cell activation, but silencing or genetic deletion of this isoform alone is insufficient. They indeed evidenced that DGKα pharmacological inhibition resulted in an improvement of Ras/ERK signaling and AP‐1 transcription and the enzyme localization at the plasma membrane, postulating that inhibitor binding results into enzyme membrane localization in a conformation that increases its scaffolding functions and TCR responses. DGKα silencing instead impaired Lck (lymphocyte‐specific protein tyrosine kinase) activation resulting in limited costimulation responses and suggesting a kinase‐independent scaffolding function.

Although both R59949 and R59022 have demonstrated to be nontoxic for noncancerous cells at the doses that affect cancer cell viability in vitro, they resulted to be nonoptimal for the in vivo use, with a predicted poor blood brain barrier penetration and a short half‐life [146].

**Figure 4:**
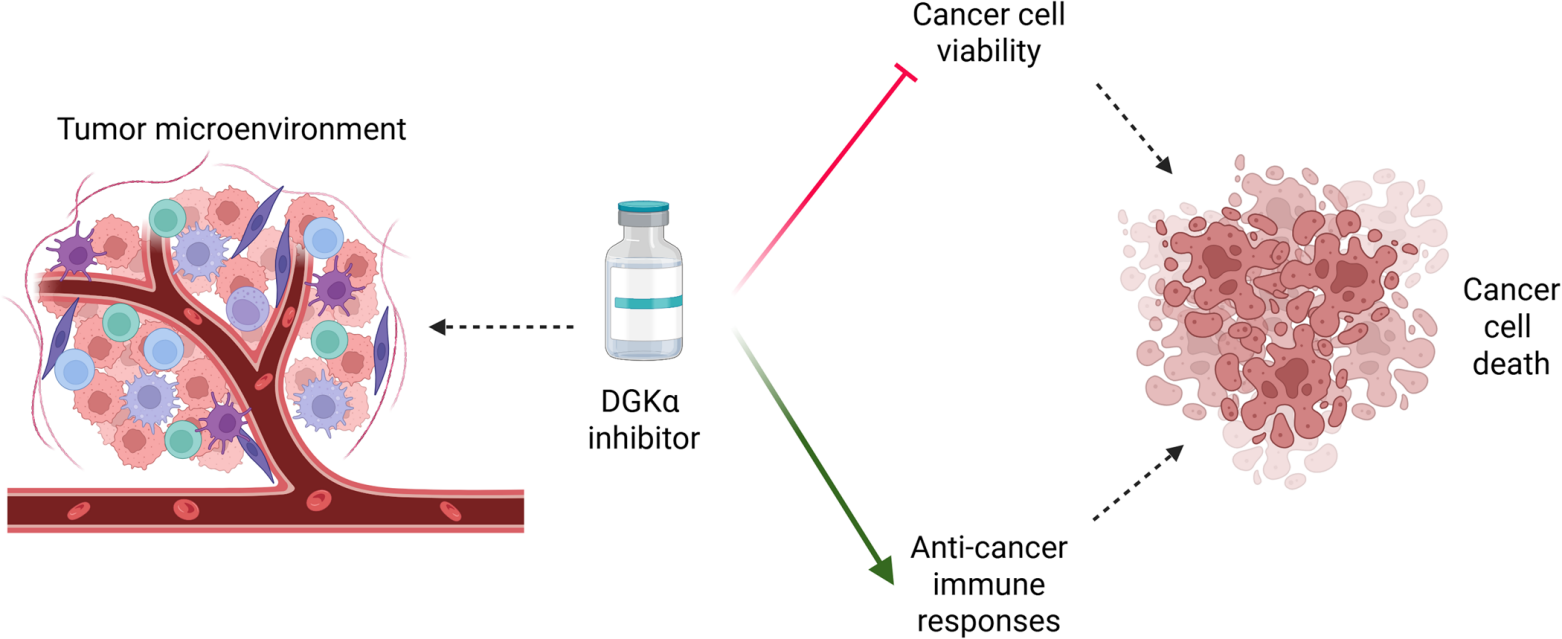
The double efficacy of DGKα inhibitors on cancer cells. The addition of DGK inhibitor negatively affects cancer cell viability and improve anticancer immune responses, potentiating the therapeutic efficacy. Created with BioRender (https://www.biorender.com/). [Color figure can be viewed at wileyonlinelibrary.com]

The research for DGK‐isoform specific inhibitors focused mostly on the α isoform, probably because it is one of the most characterized and its role in physiopathological processes has been extensively studied. In this context Boroda et al. explored the use of ritanserin for DGKα inhibition. This molecule, originally proposed for the treatment of schizophrenia and substance dependence, has indeed a structure very close to R59022 and R59949. Classified at first as a serotonin receptor antagonist (5‐HTR), ritanserin possesses good pharmacokinetic properties and causes few side effects. It demonstrated its prevalent inhibitory activity against the sole DGKα (IC_50_ value of 15 μM reported) in a dose‐dependent manner, even if growing doses are able to affect the activity also of other isoforms, and therefore its repurposing for cancer treatment was proposed. Moreover, also R59022 and R59949 resulted to be serotonin receptor antagonists [199]. For these reasons, ritanserin has been proposed for cancer treatment, for instance by Fu et al. who exploited its capability to highlight the DGKα role in non‐small cell lung cancer‐related metastasis in vitro and in vivo [34]. Tan et al. otherwise exploited ritanserin to pharmacologically inhibit DGKα in acute myeloid leukemia, observing a decreased cell proliferation and increased apoptosis in vitro, as well as efficacy as anti‐cancer drug in vivo models. This treatment indeed negatively regulates Jak‐Stat and MAPK signaling pathways, as well as SphK1 expression through DGKα inhibition [200]. Ritanserin has also been proposed for the treatment of glioblastoma by Olmez et al. who demonstrated that the mesenchymal subtype was particularly susceptible to this treatment, because DGKα inhibition resulted in the inhibition of geranylgeranyltransferase I, perturbing its downstream mediators, such as NF‐κB [217]. The exact mechanism for DGKα inhibition is still unclear, but it was demonstrated that ritanserin interacts with both C1 and catalytic accessory domains, rather than the ATP binding pocket [20]. Since both ritanserin and R59022 affect Type I DGKs activity at the 50 μM dose, Granade et al. searched an alternative specific for the α isoform between a library of 188 Ritanserin analogues, selecting among others the compound JNJ‐3790339 (DGKα IC_50_: 9.6 μM), as it resulted to be more isoform specific compared to other molecules and additionally resulted to be more cytotoxic for cancer cells, namely a melanoma, a glioblastoma multiforme and malignant T cell lines, and was able to enhance T cell activation [201], as exemplified in Figure 4.

Velnati et al. performed a deep virtual screening to find new DGKα‐specific inhibitors for the treatment of X‐linked lymphoproliferative disease. They exploited the well‐known inhibitors R59949 and R59022 as templates to discover new putative DGKα inhibitors based on their structural similarity with these two molecules. AMB639752 emerged as an optimal candidate, because it was able to selectively inhibit the α isoform activity with an IC_50_ of 4.3 μM, and moreover avoided the off‐target effect on serotonin receptors. They tested therefore AMB639752 on several models, comparing its effects especially with those obtained with the administration of ritanserin, observing an increased response not due to the involvement of the serotonin‐related signaling [184]. The same group analyzed later the ability of AMB639752 derivates in inhibiting DGKα activity, perturbing serotonin receptors, rescuing the reactivation‐induced cell death in X‐linked lymphoproliferative disease models and reducing breast cancer cell migration. They found again some promising candidates, such as compounds 11 and 20 which with an IC_50_ respectively of 1.6 and 1.8 µM for the isoform of interest, moreover, generating a pharmacophore model for DGKα‐specific inhibitor design [202].

Otherwise, the high throughput screening performed by Liu et al. allowed the identification of CU‐3 as DGKα‐specific inhibitor of (0.6 μM IC_50_ measured by a modified octyl glucoside mixed micellar DGK activity assay) among other 9600 chemical compounds. Further analysis identified its target within the enzyme catalytic domain, where CU‐3 competitively inhibits the affinity of DGKα for ATP, and validated its potential for improving cancer cell apoptosis and increasing IL‐2 production in T cells, suggesting a dual role in cancer therapy for this compound. Before focus their attention on CU‐3 they also screened other molecules, namely CU‐1, CU‐2, and CU‐4, and found that although they principally inhibit the α isoform, they also partially affect other isoenzymes, such as DGKη by CU‐1, or DGKδ, DGKκ, and DGKι by CU‐4 [203]. The same group continued this study by identifying another specific inhibitor, namely Compound A, which limited the kinase activity more effectively than CU‐3, but also affected the β and γ isoforms (IC_50_ value for DGKα: 0.04 μM, for DGKβ: 0.02 μM, for DGKγ: 0.01 μM). They reported that both CU‐3 and Compound A increased IL‐2 production in T cells and provoked cell death in melanoma and several other cancer cells [204]. Possessing similar features, the semi‐selective DGKα inhibitor DGKAI (declared IC_50_ α:0.01, β: 0.01, γ: < 0.01 μM) was effective on hepatocarcinoma inhibition and T cell immune responses enhancement in vivo, maximizing the therapeutic effects when given in combination with anti‐PD‐L1 monoclonal antibody [205].

Another α‐isoform specific inhibitor, called ISM4312A, was recently presented by Sun et al. who demonstrated its efficacy in a MC38 in vivo syngeneic model, where it showed significant antitumor properties with or without an anti‐PD‐1 combined treatment. The reported IC_50_ is particularly low: 120 pM [206].

Very recently, Antypenko et al. performed an in‐depth computational analysis to identify novel molecules potentially able to influence DGKα activity. Starting from patent CN 115362003 B, which presents the [1,2,4]triazolo[1,5‐c]quinazoline scaffolds as platform to build DGK modulators, the authors explored spiro‐fused analogues, discovering some candidates with a promising therapeutic potential [218].

Hattori et al. instead focused on DGKγ, one of the isoforms localized in the brain, which does not have a selective inhibitor, and furthermore its functions are still under investigation. They exploited a positron emission tomography (PET) technique to assess DGKγ localization to monitor its activity in the brain. Evaluating 3‐acetyl indole derivatives as probes suitable for this purpose, they also tested their capability of kinase inhibition. They discovered that 6‐Methoxypyridine derivative 9 was a suitable as PET probe, even if it also possessed a potential as inhibitor (IC_50_ 30 nM) while N‐methyl derivative 2 emerged as inhibitor, with an IC_50_ value of 13 nM for DGKγ [207].

As mentioned earlier, Hayashi et al. discovered that the KU‐8 compound could activate DGKα. However, at the same dose (100 μM), it inhibited several other isoenzymes, particularly DGKκ (less than 20% activity compared to control), as well as DGKι (30%), DGKβ (50%), DGKη (80%), and DGKγ (80%). Therefore, a careful use of this molecule should be done, because it can differently influence the activity of different DGKs [183].

The case of DGKζ is quite different, because very few proposals are deposited in the literature. Instead, it is possible to find several compounds by searching on clinical trials and patents databases. An example of DGKζ inhibitor is presented by Okumura et al. who evaluated for this purpose a pyridazinyl thiazole‐carboxamide compound, that is, ASP1570, in natural killer cells. They found out that the blocking of this DGK isoform achieved by ASP1570 improved ERK phosphorylation, IFNγ production, and degranulation of immunoreceptor‐activated natural killer cells in vitro and in vivo, where tumor clearance was also enhanced [208]. Surprisingly, this compound promotes DGKζ proteasome‐mediated degradation, thus promoting DAG accumulation but also removing eventual scaffolding functions of the protein. This action releases tumor‐infiltrating T cells from the anergic state and counteracts multiple inhibitory receptors signaling, inhibiting tumor growth in murine models [209].

BAY 2965501 from Bayer is another proposal for DGKζ inhibition. This compound is commercially available, but the selectivity against other DGK isoforms is not explicated. However, it is reported its high selectivity on the ζ isoform (reported IC_50_ in WO2021214019 of 4.62 μM) and the enhanced tumor cell killing mediated by T cells and natural killer cells after its administration, despite not directly reducing cancer cell proliferation in vitro. Furthermore, BAY 2965501 treatment in vivo reduced tumor growth and enhanced anti‐PDL‐1 antibody outcomes [210, 211].

Interestingly, Li et al. presented another possible way to achieve ζ inhibition studying the protective effect of momordicine I, a compound extracted from *Momordica charantia* L., on isoproterenol‐induced hypertrophy in vitro. They indeed highlighted its efficacy in mitigating cardiomyocyte hypertrophy (dose 12.5 μg/mL) and reducing DGKζ and glycerophospholipid metabolizing enzymes group VI phospholipase A_2_ mRNA and protein expression induced by the isoproterenol treatment, but the mechanism of action has not been yet completely understood and further studied are needed, even if this molecule is commercially available as DGKζ inhibitor [212] and has been employed for the experimental treatment of head and neck cancer [219].

Other efforts have been spent to find dual specificity inhibitors targeting both α and ζ isoforms for antitumor purposes. In this context, some researchers developed a process to synthetize naphthyridinone derivatives with this specific property, finding several molecules selective to one or both isoforms [220]. Indeed, they refined a phenotypic screening on T cells to select molecules able to block intracellular checkpoint signaling. Starting from the quinolone 1 (BMS‐684), they tested other derivates with different chemical modifications, finally developing BMS‐502, that had an inhibitory activity against DGKα, ζ and ι, with a reported IC_50_ of 0.0046, 0.0021 and 0.0026 μM for the three isoenzymes respectively, while BMS‐684 affected principally the α activity (DGKα IC_50_ value of 0.015 μM). Comparative studies in vitro of BMS‐502 and other inhibitors, as ritanserin and R59949, showed that this compound induced and enhanced immune responses due to an improved inhibitory activity. Furthermore, BMS‐502 featured very promising pharmacokinetics properties, which make it a promising candidate for the clinic [213]. In addition, other molecules emerged from further studies, as BMS‐332, specific for the α and ζ, also hitting the ι isoforms with a reported IC_50_ of 0.009, 0.008, and 0.01 μM, respectively [214]. Finally, a BMS DGKα/ζ double inhibitor of the BMS series (most likely one of those presented before) was recently able to potentiate antitumor immunity in mouse models, also synergizing with the PD‐1 blockade [221]. Bristol‐Myers Squibb Company holds numerous patents with BMS compounds and their applications (WO2020006016, WO2020006018, WO2022187406, WO2021041588, WO2024054944).

Tu‐Sekine et al. instead focused their attention on DGKθ. Exploring some regulatory pathways of this isoform and its activity in the nucleus, they also monitored the effects of some commonly used pharmacological inhibitors on DGKθ activity, because these molecules were often exploited to study nuclear pathways, and they wanted to check their effect on this enzyme. Surprisingly, they observed how some of them, as U73122 a PI‐PLC inhibitor, was able to negatively modulate also the kinase activity competing for the substrate, thus the results obtained with these molecules should be interpreted with cautions, because they are able to interfere also with this kinase, influencing the phenomenon studied [215].

## 6 Clinical Trials With DGK Inhibitors

Currently, several clinical trials are investigating the use of DGKα or DGKζ inhibitors either as monotherapy or in combination with other cancer treatments. The rationale is that inhibiting DGK enzymes could have a dual beneficial effect: potentiating antitumor immune responses while simultaneously directly targeting cancer cells [31]. Furthermore, studies have shown that DGK inhibitors can enhance the effects of immune checkpoint inhibitors, such as anti‐programmed cell death‐1 (PD‐1) and anti‐PD‐1 ligand (PD‐L1) antibodies [31]. Cancer cells can exploit the PD‐1/PD‐L1 axis, which diminishes TCR responses, to evade immune system action. However, the efficacy of these treatments is limited, with clinical response rates ranging from 10% to 40% across various cancers [222]. Because DGKα and DGKζ negatively regulate TCR, inhibiting them can enhance T cell antitumor responses and improve the efficacy of PD‐1/PD‐L1 therapy [214].

The results of these trials will be a significant milestone in the field, providing valuable insights into the efficacy of this strategy for treating the condition. The active clinical trials involving DGK inhibitors are listed below in Table 6.

**Table 6:** Clinical trials involving DGK inhibitors.

Clinical trial	Inhibitor	Inhibited isoform	Key information	References
NCT05858164	BAY2862789	DGKα	A phase 1 study to evaluate safety, tolerability, dose, pharmacokinetics, pharmacodynamics, and tumor response upon inhibitor treatment in participants with advanced solid tumors	[223]
NCT06082960	GS‐9911	DGKα	A phase 1 study to evaluate the safety of GS‐9911 as monotherapy and in combination with Zimberelimab in advanced solid tumors	[224]
NCT05083481	ASP1570	DGKζ	A phase 1/2 study to evaluate ASP1570 as monotherapy and in combination with Pembrolizumab or standard therapies including chemotherapy and/or immunotherapy in advanced or metastatic solid tumors	[225]
NCT05614102	BAY2965501	DGKζ	A phase 1 study to assess safety, tolerability, dose, pharmacokinetics, pharmacodynamics, and tumor response profile of the inhibitors as monotherapy or in combination with Pembrolizumab and platinum‐based chemotherapy, in participants with advanced solid tumors	[226]
NCT05904496	BGB‐30813	DGKζ	A phase 1a/1b study to investigate the safety, tolerability, pharmacokinetics, pharmacodynamics, and antitumor activity of the inhibitor as monotherapy or combined with Tislelizumab in patients with advanced or metastatic solid tumors	[227]
NCT05407675	BMS‐986408	DGKα, DGKζ	A phase 1/2 study to evaluate BMS‐986408 alone and in combination with Nivolumab or with Nivolumab and Ipilimumab in advanced solid tumors	[228]
NCT06873789	INCB177054	DGKα, DGKζ	A phase 1/2 study of INCB177054 in participants with select advanced or metastatic solid tumors	[229]

First of all, there are two clinical trials involving DGKα inhibitors. One is the clinical trial NCT05858164 involving BAY2862789 (Bayer): the aim is to use this molecule to potentiate T cell responses against cancer cells, especially in solid tumors as non‐small cell lung carcinoma. In this case, the inhibitor is administered alone to elucidate its safety and pharmacokinetics properties [223]. Both Gilead Sciences and Bayer currently market DGKα‐specific inhibitors under patents (WO2022271650, WO2022271659, and WO2022271684 by Gilead Sciences; WO2021105115 and WO2021105117 by Bayer). Another example is the NCT06082960 trial, which is expected to conclude by the end of 2026. This trial is evaluating the efficacy of the small molecule GS‐9911 (Gilead Sciences) as a DGKα inhibitor for treating advanced solid tumors in adults, either as monotherapy or in combination with the anti‐PD‐1 monoclonal antibody Zimberelimab. The structure of this compound has not been shared [224].

In the case of DGKζ conversely three clinical trials are ongoing. The study of Okumura et al. on ASP1570 indeed paved the way for the clinical trial NCT05083481, that aims to verify the efficacy of this molecule, given alone or in combination with Pembrolizumab, a monoclonal antibody against the PD‐1 receptor, to burst T cell responses against solid tumors in adults, or standard therapies including chemotherapy and/or immunotherapy, after a first phase to determine the optimal dose for further treatment. The results will be ready in 2028 [225]. Another trial otherwise involves BAY 2965501, which is now under investigation in the trial NCT05614102 for the treatment of solid tumors, in particular in the skin, kidney, lung, and stomach, as monotherapy or in combination with Pembrolizumab again. This trial aims indeed to potentiate T lymphocytes activity against cancer cells, and is estimated to be completed by 2027 [226]. The DGKζ inhibitor BGB‐30813, developed by BeiGene, is currently under clinical trial (NCT05904496) to evaluate its safety, pharmacokinetics, pharmacodynamics, and antitumor efficacy given alone or with tislelizumab (PD‐1 antibody) in solid tumors or metastasis. The results are expected to be available by 2026 [227]. The selectivity of this compound against other DGK isoforms, especially DGKα, is not reported, but since the patent (WO2023125681) contains detailed information about the IC_50_ of every compounds with both DGKζ and DGKα, it is probably that they chose the higher selective molecules for the clinical trial.

Moreover, there is one clinical trial (NCT05407675) involving a BMS‐series molecule, namely BMS‐986408, which aims to determine its maximum tolerated dose as monotherapy and in combination with nivolumab or nivolumab and ipilimumab, further establishing the pharmacokinetic and pharmacodynamic in patients with advanced solid tumors. The results will be available from October 2025. BMS‐986408, already commercialized as “DGK inhibitor,” is most likely one of the DGKα and ζ double inhibitors, as reported in Scifinder searching for its CAS number (2618418‐12‐3) [228].

A new trial (NCT06873789) involving a DGKα and ζ double inhibitor (INCB177054 [230], Incyte Corporation) is currently starting. It will evaluate the efficacy of this molecule given as monotherapy or in combination with retifanlimab in participants with select advanced or metastatic solid tumors. The results will be available in 2028 [229].

## 7 Conclusions

In summary, DGKs are a family comprising 10 different enzymes where every member has distinct structural and functional properties and features, even if several isoforms could co‐exists in the same biological system ensuring a certain degree of redundancy. While there is growing evidence of their involvement in various physiological and pathological processes, including cancer, significant knowledge gaps remain. One such gap is the lack of an experimentally determined 3D structure, likely due to technical challenges with crystallizing lipid kinases, which may be overcome by advancements in techniques such as cryo‐electron microscopy. A deeper understanding of their structural features could enable rational drug design, allowing the virtual reconstruction of the target, identification of binding pockets, and selection of potential ligands through molecular docking analysis.

At present the search for new molecules able to modulate their activity can be based on structure‐based drug design or ligand‐based drug design [231, 232]. In the first case, the target is reconstructed and the affinity with known ligands exploited to identify binding pockets; the resulting information of the target, as well as ligands, are used to design new ligand molecules with the required profile. In the second case, the structural information and physicochemical properties of active and inactive ligands are examined to determine the features required for the new drugs. Both these methods can be adopted for DGK inhibitor search for instance, because known modulators could be exploited to validate structure‐based drug design protocols, also if the enzyme structure is almost entirely predicted, and additionally the ligand similarity could be checked. Alternatively the development of high throughput activity assays such as the luciferase assays commercially available (Promega) enable library screening followed by compound refinement. In the case of DGKα, for example, searches based on R59949 and R59022 resulted in the selection of ritanserin and other compounds quite isoform‐specific, while high throughput screening resulted in CU‐3 that is very active but never used in vivo. A more challenging situation emerges with the less explored DGKs, because for longer isoforms also AlphaFold predictions become extremely unreliable and template compounds are few or absent.

Currently, there are some clinical trials involving DGKα or DGKζ, assessing their pharmacological properties and efficacy for their use as anticancer agents. Surely the results will give information about the feasibility of this therapeutic approach and, if successful, will pave the way for further investigations not only in the cancer field. Indeed, DGKs play an important role in other fields relevant for translational medicine such as the immune system. It would be also extremely interesting to monitor the presence of eventual side effects correlated to the administration of these drugs, to introduce eventual strategies to directly target the cells of interests.

Thus, we believe that the development of DGK isoform‐specific targeting molecules is a field approaching maturity and with the possibility to expand beyond oncology. For this purpose, a better understanding of the role that each isoenzyme plays will help to select the specific pathologies associated with an abnormal activity of each family member and to reveal the translational opportunities.

## Data Availability

Data sharing not applicable to this article as no datasets were generated or analysed during the current study.
